# Associations between psychiatric diagnoses in parents and psychiatric, behavioral, psychosocial outcomes in their offspring: a Swedish population-based register study

**DOI:** 10.1192/j.eurpsy.2024.202

**Published:** 2024-08-27

**Authors:** M. Zhou, C. T. Lageborn, A. Sjölander, H. Larsson, B. D’Onofrio, M. Landén, P. Lichtenstein, E. Pettersson

**Affiliations:** ^1^Karolinska Institutet, Stockholm, Sweden; ^2^Indiana University, Bloomington, United States

## Abstract

**Introduction:**

Children with parents with psychiatric diagnoses have an increased probability for not only the same condition as their parent, but also for other conditions and behavioral and psychosocial problems. Whereas many studies have focused on parental severe mental illness due to their significant impairment, less attention has been paid to more common disorders despite their higher prevalence. In addition, because most past research only included one exposure or one outcome at a time, it remains difficult to examine and compare broad patterns of intergenerational transmission.

**Objectives:**

To examine associations between six parental psychiatric diagnoses in parents, and a broad range of psychiatric diagnoses, psychotropic medications, criminality, suicide, violent victimization, accidents, and school and labor performance in their offspring.

**Methods:**

Based on Swedish national registers, we linked all individuals born in Sweden between 1970 and 2000 to their biological parents (*N* = 3 286 293). We used a matched cohort design, analyzed with stratified Cox regression and conditional logistic regressions to examine associations between six psychiatric diagnoses in the parents, and 32 outcomes in their offspring. All exposed and unexposed children were followed from their date of birth to the date of emigration from Sweden, the death, or 31 December 2013 when the offspring were 14-44 years old.

**Results:**

In terms of absolute risk, most children who had parents with psychiatric diagnoses were not diagnosed in specialist care themselves, as the proportion of having any of the 16 types of psychiatric conditions ranged from 22.17% (exposed to parental depression) to 25.05% (exposed to parental drug-related disorders) at the end of follow-up. Nevertheless, in terms of relative risk, all six parental psychiatric diagnoses increased the probability of all 32 outcomes in their offspring, with the Hazard Ratio ranging from 1.04 to 8.91 for time-to-event outcomes, and the Odds Ratio ranging from 1.29 to 3.36 for binary outcomes. Some specificities were observed for parental psychotic and substance misuse diagnoses, which strongly predicted offspring psychotic-like and externalizing-related outcomes, respectively.

**Conclusions:**

The intergenerational transmission of parental psychiatric conditions appeared largely transdiagnostic, even for non-psychiatric outcomes in offspring. Given the broad spectrum of associations with the outcomes, service providers (e.g., psychiatrists, teachers, and social workers) should consider clients’ broader psychiatric family history when predicting prognosis and planning interventions/treatment.

**Disclosure of Interest:**

None Declared

